# Choline Supplementation in Pregnancy: Current Evidence and Implications

**DOI:** 10.7759/cureus.48538

**Published:** 2023-11-08

**Authors:** Arpita Jaiswal, Deepika Dewani, Lucky Srivani Reddy, Archan Patel

**Affiliations:** 1 Obstetrics and Gynaecology, Jawaharlal Nehru Medical College, Datta Meghe Institute of Higher Education and Research, Wardha, IND

**Keywords:** cognitive outcomes, neural tube defects, maternal health, fetal development, pregnancy, choline supplementation

## Abstract

Choline, an essential nutrient, is pivotal in supporting maternal and fetal health during pregnancy. In this comprehensive review, we explore the current evidence and the implications of choline supplementation in pregnancy. Choline is indispensable for neural tube formation, brain development, and the overall well-being of expectant mothers, rendering it a cornerstone of prenatal care. Inadequate choline intake is associated with neural tube defects, cognitive deficits in offspring, and maternal health complications. This review highlights the potential benefits of choline supplementation as demonstrated by clinical studies, including the reduction of congenital disabilities and improvements in cognitive outcomes. However, it is important to acknowledge the presence of methodological challenges, ethical considerations, and variations in study findings. To harness the full potential of choline in maternal nutrition, we advocate for increased awareness, continued research, and the development of ethical guidelines. In the broader context, policymakers must consider integrating choline recommendations into dietary guidelines. This step would ensure equitable access to this vital nutrient for all pregnant women, regardless of socioeconomic status. In conclusion, choline supplementation shows promise in enhancing both maternal and fetal health. However, its full benefits can only be realized through further research and concerted efforts in raising awareness.

## Introduction and background

Choline is an essential nutrient that plays a pivotal role in various physiological processes, and its significance during pregnancy cannot be overstated. Choline is a water-soluble amine that serves as a precursor for phospholipids, which are integral components of cell membranes. Furthermore, it is a vital component of acetylcholine, a neurotransmitter involved in numerous brain functions. Choline is particularly critical during pregnancy due to its multifaceted contributions to maternal and fetal health [1].

During pregnancy, the demand for choline significantly increases as it is involved in vital processes such as neural tube formation, brain development, and the synthesis of lipoproteins. It is also essential for liver function and lipid metabolism. Choline's significance in pregnancy is underscored by its potential to prevent neural tube defects (NTDs), enhance cognitive development in the offspring, and promote overall maternal well-being [2].

The rationale for examining choline supplementation during pregnancy is multifaceted. While choline is crucial for fetal development, studies suggest that many pregnant women do not meet the recommended dietary intake, potentially risking maternal and fetal health. This discrepancy between choline requirements and actual intake raises questions about the need for supplementation and its potential benefits [3].

Additionally, recent research has indicated that choline supplementation might have far-reaching implications for prenatal and postnatal health, including preventing cognitive deficits, improving birth outcomes, and possibly reducing the risk of certain developmental disorders. However, the existing body of evidence on the subject is diverse and sometimes contradictory, necessitating a comprehensive review to synthesize the current knowledge [4].

This review aims to provide an in-depth examination of the current evidence surrounding choline supplementation during pregnancy. We aim to evaluate the role of choline in maternal and fetal health, explore the consequences of choline deficiency, and assess the effectiveness and safety of choline supplementation. Furthermore, we seek to identify the implications of choline supplementation for healthcare practices, public policy, and future research.

## Review

Choline in pregnancy

Role of Choline in Fetal Development

Choline's significance in fetal development is indeed noteworthy. Its role as a precursor to phosphatidylcholine, a crucial component of cell membranes, makes it indispensable for cellular growth and division. The impact of choline begins very early in pregnancy, potentially before the mother is aware of her condition. This underscores the necessity for adequate choline intake before and during the initial stages of pregnancy [1]. Notably, choline's influence extends beyond the development of the neural tube; it also contributes to the formation of the fetal brain and the establishment of neural connections. Research indicates that choline intake during pregnancy may have enduring effects on the cognitive function, memory, and behavior of the offspring. Moreover, choline plays a critical role in preventing fatty liver disease, a condition that can arise during pregnancy and have adverse implications for both the mother and the developing baby [5].

Choline Sources in the Diet

Choline is not a nutrient that the human body can synthesize in sufficient quantities; therefore, it must be obtained from dietary sources. Several foods provide choline, making it accessible through a balanced diet. These sources are mentioned in Table 1 [6].

**Table 1:** Choline sources in the diet

Dietary Source	Description
Eggs	Egg yolks are one of the richest dietary sources of choline, with approximately 125 milligrams per large egg. They are an accessible and versatile option for increasing choline intake.
Meat and Poultry	Beef, chicken, and pork are excellent sources of choline, and their choline content varies based on the cut and preparation. Incorporating lean cuts of meat into one's diet significantly contributes to choline intake.
Fish	Certain types of fish, such as salmon and cod, are known for their choline-rich content. They not only provide an abundance of choline but also essential omega-3 fatty acids, making them a wholesome choice, especially for pregnant women.
Dairy products	Dairy items like milk, yogurt, and cheese offer choline. The exact choline content may vary between products. These dairy sources are commonly consumed and provide multiple essential nutrients and choline.
Legumes and nuts	Plant-based sources of choline include soybeans and peanuts. These legumes and nuts contribute choline and health benefits such as protein, fiber, and healthy fats.
Cruciferous vegetables	Vegetables such as broccoli and Brussels sprouts contain choline, albeit in smaller quantities than other sources. They are a valuable addition to a balanced diet, offering a range of nutrients.
Whole grains	Certain whole grains, such as quinoa, contain moderate amounts of choline. Whole grains are essential for their fiber content and provide a range of nutrients, making them a valuable part of a varied diet.

Choline Requirements During Pregnancy

Choline requirements during pregnancy significantly exceed those in non-pregnant periods. According to the Recommended Dietary Allowance (RDA) set by the National Academy of Medicine, these requirements vary based on the stage of pregnancy. In the early pregnancy phase, spanning up to 12 weeks, the recommended choline intake stands at 450 milligrams per day, and it escalates to 550 milligrams per day during the later stages of pregnancy, after 12 weeks. These RDA values notably surpass the daily choline intake suggested for non-pregnant women, which hovers around 425 milligrams. Meeting these augmented choline needs is paramount to ensure optimal fetal development and maternal well-being. Nevertheless, many women struggle to attain these levels solely through their diet, which has spurred discussions about the potential advantages of choline supplementation. In the ensuing sections of this review, we will delve into the repercussions of choline deficiency and scrutinize the existing evidence concerning choline supplementation during pregnancy [7].

Choline deficiency and risks

Consequences of Choline Deficiency in Pregnancy

Neural tube defects: Inadequate choline intake during pregnancy poses a heightened risk of NTDs in the developing fetus. Choline is a critical factor in early neural tube formation, and its insufficiency can result in NTDs such as spina bifida. These debilitating congenital conditions affect the spinal cord and brain development, underscoring the importance of choline in preventing such devastating congenital disabilities [8].

Impaired brain development: Choline is an indispensable nutrient for adequately developing the fetal brain. Its role in cell membrane formation and neurotransmitter synthesis is pivotal, and a deficiency during pregnancy may lead to long-term cognitive deficits in the child. Evidence suggests that insufficient choline intake could increase the child's susceptibility to neurodevelopmental disorders, emphasizing the significance of choline in fostering healthy brain development [9].

Liver function: Choline plays a crucial role in maintaining liver health. A choline deficiency can lead to the development of fatty liver disease, a condition that has significant implications for both the expectant mother and the developing fetus. Fatty liver disease is characterized by fat accumulation in liver cells, which can ultimately result in liver dysfunction. This, in turn, poses a substantial risk to the overall well-being of both the mother and the fetus [10].

Cognitive and behavioral issues: Some research findings indicate a potential link between inadequate choline intake during pregnancy and cognitive and behavioral issues in the offspring. It is worth noting that the assessment of choline's impact on offspring's cognitive and behavioral development can be conducted through the examination of both maternal diet history and supplementation history [11]. Children exposed to lower choline levels in utero may be at greater risk of experiencing challenges in learning, memory, and overall behavior, further underscoring choline's role in promoting healthy cognitive development [11].

Increased maternal health risks: Choline deficiency poses a significant concern for both the developing fetus and the expectant mother. This deficiency can lead to a heightened risk of maternal complications, including conditions such as hypertension and preeclampsia. These maternal health complications not only adversely affect the well-being of the mother but also have potential repercussions on the developing baby's health [1]. This underscores the critical relationship between choline deficiency and maternal health, emphasizing the importance of addressing it for the benefit of both the mother and the developing fetus.

Factors Contributing to Choline Deficiency

Dietary choices: Choline deficiency during pregnancy can often be attributed to poor dietary choices. A lack of choline-rich foods in one's diet is a primary factor. Those following vegetarian or vegan diets, which typically exclude animal products high in choline, may be particularly at risk. With careful planning and including plant-based sources of choline, individuals on these diets may be able to meet their choline requirements, potentially putting both maternal and fetal health at risk [12].

Morning sickness: Severe morning sickness, a common issue during pregnancy, can exacerbate choline deficiency. This is because morning sickness can lead to decreased food intake, making it challenging for expectant mothers to consume adequate choline. The lack of nutritional intake, including choline-rich foods, can contribute to a shortfall in this essential nutrient [13].

Limited awareness: Limited awareness about the importance of choline in pregnancy is another contributing factor. Many expectant mothers may need to be fully informed about the role of choline in fetal development and maternal health. As a result, they may not actively seek out or prioritize choline-rich foods in their diets. Increasing awareness and education about the significance of choline during pregnancy is crucial to address this issue [1].

Malabsorption: Certain medical conditions or medications can hinder the absorption of choline in the body. Conditions affecting the gastrointestinal tract, liver, or gallbladder can reduce choline absorption, leading to lower availability. Medications that interfere with choline metabolism can also contribute to choline deficiency. Identifying and managing such conditions and medications is essential for preventing deficiency [14].

Individual variability: Choline metabolism is subject to individual variability. Genetic factors and metabolic differences can influence how efficiently choline is processed and utilized in the body. Some individuals may have higher or lower choline needs based on their genetic makeup, potentially leading to variations in choline requirements. Recognizing and accommodating this variability is essential to ensure that pregnant women receive the appropriate amount of choline to support maternal and fetal health [15].

Prevalence of Choline Deficiency in Pregnant Women

Determining the prevalence of choline deficiency in pregnant women can be challenging, but various studies have shed light on the issue. Research has indicated that many pregnant women do not meet the recommended choline intake levels. The prevalence of deficiency can vary by region, dietary habits, and population demographics [12]. To address this concern, it is essential to assess the dietary habits of pregnant women and understand the factors contributing to choline deficiency. This information can guide healthcare professionals and policymakers in developing strategies to promote adequate choline intake during pregnancy, potentially mitigating the associated risks and complications [16].

Choline supplementation

Forms of Choline Supplements

Choline bitartrate: Choline bitartrate is a widely used choline supplement. It is often chosen to increase choline intake and is readily available in many over-the-counter products. Choline bitartrate is appreciated for its cost-effectiveness and ease of accessibility, making it a common choice for individuals looking to address choline deficiency or support their overall health [6].

Phosphatidylcholine: Phosphatidylcholine is a type of choline found in dietary sources such as soy lecithin. It is recognized for its role in liver health and is available in supplement form. Phosphatidylcholine is sometimes used to promote liver function and protect against conditions such as fatty liver disease, making it valuable for individuals seeking to support their liver health [2].

Choline chloride: Choline chloride is a highly concentrated source of choline and is often used in animal feeds to ensure optimal growth and development in livestock. It is also available in some dietary supplements, offering a concentrated source of choline for those with specific dietary or health needs. Its supplement use is typically tailored to meet precise choline requirements [17].

Alpha-GPC (L-alpha glycerylphosphorylcholine): Alpha-GPC is a choline compound that has gained attention for its potential cognitive-enhancing effects. It is commonly used as a nootropic supplement and is believed to enhance memory, attention, and cognitive function. Alpha-GPC is a popular choice for individuals seeking cognitive support and is often found in products targeting cognitive enhancement [18].

CDP-choline (cytidine 5'-phosphocholine or citicoline): CDP-choline is another choline supplement known for its potential cognitive benefits. It is sometimes prescribed in clinical settings for neurological conditions such as stroke recovery or cognitive impairment. CDP-choline is valued for its neuroprotective properties and role in promoting acetylcholine synthesis, a neurotransmitter critical for memory and learning [19].

Choline Supplementation Guidelines

Individual nutrient needs: The nutrient requirements of pregnant women can vary significantly from person to person due to factors such as age, pre-existing dietary habits, and underlying medical conditions. It is paramount for pregnant women to consult with their healthcare providers to determine their specific nutrient needs, including choline. Healthcare professionals can assess an individual's overall health and lifestyle to provide personalized choline intake and supplementation guidance. This personalized approach ensures pregnant women receive the right amount of choline to support their unique circumstances [20].

Balanced diet: While choline supplementation may be necessary in some cases, it should complement, not replace, a balanced diet. A well-rounded and nutritious diet that includes a variety of choline-containing foods remains essential for pregnant women. Choline-rich dietary sources provide choline and a range of other vital nutrients. Incorporating foods such as eggs, meat, dairy products, and vegetables into their daily meals can help pregnant women meet their choline requirements while reaping the benefits of a diverse and wholesome diet [21].

Safety: Choline supplements are generally considered safe when used as directed, adhering to recommended dosages. However, pregnant women must follow the guidance of their healthcare providers or nutritionists when considering choline supplementation. Excessive choline intake can lead to potential side effects or adverse reactions, such as gastrointestinal distress or a fishy body odor. To ensure safety and efficacy, pregnant women should avoid self-prescribing supplements and seek professional advice to determine the appropriate form and dosage of choline supplementation for their specific needs [22]. Choline supplements are generally regarded as safe when taken within recommended dosages. However, like any supplement, they can cause side effects if consumed excessively. Common side effects may include gastrointestinal discomfort such as nausea and diarrhea [21]. While the risk of choline toxicity from dietary sources is considered low, excessive use of certain supplements, particularly in doses significantly higher than recommended, can lead to choline overload, resulting in a fishy odor in the breath, sweat, and urine. Healthcare providers should be consulted to ensure that supplementation is appropriate and safe for everyone [21].

Monitoring: Regular monitoring of choline levels and overall health is essential when contemplating supplementation. Healthcare providers can conduct choline level assessments to gauge whether supplementation is necessary and to adjust dosage as needed. Monitoring overall health during pregnancy, including assessing the impact of choline supplementation, helps ensure that maternal and fetal well-being remains the top priority. This approach safeguards against potential deficiencies or excesses, promoting the best possible outcomes during pregnancy [23].

Current evidence on choline supplementation

Clinical Studies on the Effects of Choline Supplementation in Pregnancy

A subset of studies has adopted the randomized controlled trial (RCT) design, wherein pregnant women are methodically assigned to receive choline supplements or a placebo. These trials have been instrumental in probing the effects of choline on a spectrum of outcomes, encompassing critical areas such as the prevention of NTDs, regulation of birth weight, and mitigation of gestational complications. RCTs, known for their capacity to establish causal relationships, contribute significantly to understanding choline's role in maternal and fetal health. By subjecting participants to controlled interventions and random assignment, RCTs provide high-quality evidence for assessing the impact of choline supplementation during pregnancy. These studies, with their rigorous design, help establish whether the observed effects result from choline supplementation and offer insights into the potential benefits or risks [24].

Complementary to RCTs, observational studies have meticulously scrutinized the dietary behaviors of pregnant women. These investigations have sought to delineate the intricate relationship between choline intake and pregnancy outcomes. By delving into the dietary practices of expectant mothers and analyzing real-world data, observational studies offer valuable insights into the pragmatic impact of choline on maternal and fetal health. Unlike RCTs, which manipulate variables through controlled interventions, observational studies show how choline intake naturally occurs in various populations. They help researchers understand how choline intake, as it typically happens, influences maternal and fetal health. While observational studies cannot establish causality to the same extent as RCTs, they are essential for providing real-world context, identifying potential associations, and generating hypotheses for further investigation. These studies contribute to a more comprehensive understanding of the role of choline in pregnancy by exploring the dietary behaviors and outcomes of pregnant women in diverse settings [25].

Maternal and Fetal Outcomes

Neural tube defects: Choline's association with a reduced risk of NTDs is a significant finding, particularly when choline is taken during the early stages of pregnancy. NTDs, including conditions such as spina bifida, are severe congenital disabilities that affect the baby's spinal cord and brain development. Choline's role in early neural tube formation emphasizes the critical importance of ensuring adequate choline intake, as it may serve as a preventive measure against these devastating congenital disabilities. This finding has implications for prenatal care, as it underscores the potential impact of choline on the long-term health and well-being of the developing fetus [11].

Gestational complications: Some studies have suggested that choline supplementation may mitigate the risk of gestational complications, such as preeclampsia and gestational diabetes. Preeclampsia is a severe condition characterized by high blood pressure and potential damage to organs such as the liver and kidneys. Gestational diabetes, on the other hand, is a form of diabetes that develops during pregnancy. These complications can have profound effects on both maternal and fetal health. The possibility of choline supplementation reducing the risk of these complications is an encouraging development, as it can enhance overall maternal health during pregnancy and contribute to more positive pregnancy outcomes [26].

Birth weight and preterm birth: Research has explored the potential impact of choline on birth weight and preterm birth rates. Birth weight is a critical factor as it can influence the health and development of the newborn, with low birth weight being associated with a higher risk of health issues. Preterm birth, or premature birth, can also lead to various health concerns for the infant. Investigating the role of choline in regulating these factors is essential, as it may offer insights into interventions that promote healthier pregnancies and better birth outcomes. These findings can have long-lasting effects on the health and well-being of both the mother and the newborn, emphasizing the significance of choline in prenatal care [27].

Cognitive and Developmental Outcomes in Offspring

Cognitive function: Research has indicated that children exposed to higher choline levels in utero may exhibit improved cognitive function, memory, and attention later in life. Choline's role in neuronal membrane structure, neurotransmitter synthesis, and epigenetic regulation suggests that it may be vital in fostering healthy brain development. Studies have suggested that increased choline intake during pregnancy may positively influence cognitive outcomes in children, potentially enhancing their ability to learn, remember, and focus. These findings underscore the potential long-term benefits of ensuring sufficient choline intake during pregnancy [28].

Reduced risk of developmental disorders: Choline supplementation has been explored as a potential strategy for reducing the risk of developmental disorders such as autism and attention deficit hyperactivity disorder (ADHD). While the exact mechanisms are still being investigated, it is believed that choline's impact on early brain development and neuroprotection may contribute to a reduced risk of these disorders. Choline supplementation may offer a preventive approach by providing the developing brain with the necessary building blocks and protection against adverse environmental factors. The potential to lower the incidence of developmental disorders through choline supplementation highlights the significance of this nutrient in prenatal care and its broader implications for child neurodevelopment and mental health [28].

Impact on Maternal Health

Cognitive health: Choline is vital for fetal brain development and plays a role in maintaining cognitive health in adults, including pregnant women. The cognitive demands of pregnancy and the postpartum period, combined with hormonal fluctuations, can impact maternal cognitive function. Choline supplementation may offer support by ensuring that the mother's cognitive needs are met during this critical period. Enhanced cognitive function can help expectant and new mothers manage the challenges and responsibilities of pregnancy and early motherhood, contributing to their overall well-being [29].

Liver health: Choline is crucial for liver function, and its deficiency can lead to fatty liver disease, which can affect pregnant women. Fatty liver disease during pregnancy poses risks to both the mother and the developing fetus. Choline supplementation may serve as a preventive measure, helping to maintain healthy liver function and reduce the risk of liver-related complications. Supporting liver health in pregnant women is essential for ensuring their well-being and the successful progression of pregnancy [30].

Implications for healthcare and policy

Recommendations for Healthcare Providers

Education: Healthcare providers play a pivotal role in raising awareness about the importance of choline during pregnancy. They must be well informed about the significance of choline and its potential impact on maternal and fetal health. Healthcare providers should actively communicate this information to expectant mothers, emphasizing the dietary sources of choline and the option of supplementation when necessary. Education can empower pregnant women to make informed choices about their nutrition, ensuring they receive adequate choline to support a healthy pregnancy [1].

Individual assessment: Each pregnant woman is unique, with her dietary habits, lifestyle factors, and potential risk factors for choline deficiency. Conducting individual assessments of pregnant women can provide insights into their specific needs. These assessments consider dietary preferences, vegetarian or vegan diets, morning sickness, or medical conditions that might impact choline intake. By tailoring recommendations based on these assessments, healthcare providers can offer personalized guidance, ensuring that pregnant women receive the right amount of choline for their circumstances [31].

Supplementation guidance: Healthcare providers should engage in informed discussions with pregnant women about choline supplementation's potential benefits and risks. These conversations should consider the individual's specific needs and circumstances. Some pregnant women may require supplementation to meet their choline requirements, while others may obtain sufficient choline through diet alone. It is essential to discuss the appropriate forms and dosages of choline supplements if supplementation is deemed necessary. Guidance from healthcare providers ensures that expectant mothers make informed decisions that align with their health and pregnancy goals [32].

Monitoring: Integrating choline intake as a standard component of routine prenatal care is vital. Monitoring choline levels and overall health during pregnancy can help identify deficiencies or concerns promptly. Routine assessments can ensure that pregnant women consistently receive the necessary amount of choline to support their health and the optimal development of the fetus. Monitoring forms a proactive approach to addressing choline deficiencies and promoting healthy pregnancies [33].

Public Health and Policy Implications

Public awareness: Initiatives to increase public awareness about the importance of choline during pregnancy are crucial. Public health campaigns can provide expectant mothers with information about the role of choline in maternal and fetal health, as well as dietary sources of choline. These campaigns can empower women to make informed dietary choices and prioritize choline-rich foods during pregnancy. Raising awareness contributes to healthier pregnancies and better outcomes for both mothers and their babies [7].

Dietary guidelines: Policymakers and public health authorities should consider incorporating choline recommendations into official dietary guidelines for pregnant women. Clear and consistent advice on choline intake in these guidelines can provide pregnant women with practical and evidence-based dietary recommendations. This inclusion ensures that healthcare providers and expectant mothers can access authoritative guidance, fostering better compliance with choline intake recommendations [34].

Food fortification: Exploring the potential for fortifying staple foods with choline is a valuable approach to addressing choline deficiency in the population. Fortification ensures that a broader population has access to this essential nutrient, even if they may have challenges meeting their choline needs through regular dietary choices. This strategy can be especially effective in reaching pregnant women at risk of deficiency, contributing to improved maternal and fetal health outcomes [35].

Healthcare coverage: Policymakers can consider whether choline supplements should be covered by health insurance plans, particularly for pregnant women at risk of deficiency. Coverage for choline supplements can help reduce financial barriers, ensuring that those who require supplementation to meet their choline needs have access to this essential nutrient. By including choline supplements in healthcare coverage, policymakers promote equity in maternal and fetal health and support the well-being of expectant mothers [36].

Access to Choline Supplements

Availability: Choline supplements should be readily available in pharmacies and healthcare settings to provide easy access for expectant mothers. Pharmacies, clinics, and healthcare providers should stock a range of choline supplements to meet the diverse needs of pregnant women. Ensuring a consistent supply of these supplements helps guarantee that women can access them conveniently throughout their pregnancies [37].

Affordability: Efforts should be made to make choline supplements affordable and accessible to all, regardless of socioeconomic status. Ensuring affordability is vital in preventing disparities in access to essential nutrients such as choline. This can involve initiatives such as subsidies, discounts, or the inclusion of choline supplements in maternal health programs to reduce financial barriers for pregnant women [2].

Prescription and over-the-counter options: Both prescription and over-the-counter choline supplements should be considered, offering various options to meet individual needs. While some pregnant women may require choline supplementation under the guidance of healthcare providers due to specific deficiencies or medical conditions, others may opt for over-the-counter supplements as a preventive measure. Providing both options ensures that women can access the right type of choline supplement based on their unique circumstances and the recommendations of their healthcare providers [7].

Future Research Needs

Optimal timing: Research should explore the optimal timing for choline supplementation during pregnancy, considering choline's critical role in early neural tube formation. Understanding the specific windows of vulnerability and the timing of choline's influence on neural tube development is essential. This research can inform recommendations for when choline supplementation should be initiated during pregnancy to maximize its preventive effects on NTDs and other developmental outcomes [2].

Dosage and form: The most effective dosage and form of choline supplementation should be determined, considering variations in individual needs and dietary habits. Research should focus on identifying the appropriate dosage levels of choline that are safe and effective for various stages of pregnancy and for women with different dietary patterns. Additionally, investigations into the various forms of choline supplements, such as choline bitartrate, phosphatidylcholine, choline chloride, alpha-GPC, and CDP-choline, can help determine which forms offer the best bioavailability and outcomes in different situations [22].

Long-term effects: Long-term studies are needed to assess the enduring impact of choline supplementation on offspring cognitive development, behavior, and health. Investigating the cognitive, behavioral, and health outcomes of children exposed to choline supplementation during pregnancy as they grow and develop is essential. These long-term studies can provide insights into the potential lasting benefits of choline and its role in shaping the health and well-being of future generations [29].

Risk-benefit assessment: A comprehensive risk-benefit assessment should be conducted to establish clear guidelines for choline supplementation, including potential side effects and interactions with other nutrients or medications. Research should evaluate the potential risks of choline supplementation, including side effects or interactions with other substances. This assessment is crucial for developing evidence-based guidelines that help healthcare providers and pregnant women make informed decisions regarding choline supplementation [2].

Health disparities: Research should explore the potential disparities in access to choline supplements and the impact on maternal and child health, particularly among underserved populations. Investigating disparities in access to choline supplements can shed light on potential inequities in maternal and child health outcomes. Research in this area can inform strategies to ensure that all pregnant women, regardless of socioeconomic or demographic factors, have equal access to the benefits of choline supplementation [38].

Challenges and limitations

Methodological Challenges in Choline Research

Dietary assessment: Accurately measuring choline intake can be challenging due to variations in dietary reporting and the need for precise data on choline content in various foods. The accuracy of dietary assessments relies on self-reporting, which can be subject to recall bias and inaccuracies. Additionally, the choline content of foods can vary depending on factors such as cooking methods and food sources. The need for comprehensive and standardized methods for assessing choline intake is crucial for obtaining reliable data on the role of choline in pregnancy outcomes [39].

Individual variability: Choline requirements and the response to supplementation can vary significantly among individuals, making it challenging to establish one-size-fits-all recommendations. Genetic factors, metabolic differences, dietary preferences, and underlying health conditions can all influence how efficiently choline is processed and utilized by the body. This variability underscores the importance of personalized approaches to choline intake and supplementation, taking into account the specific needs of each pregnant woman [40].

Longitudinal studies: Long-term studies on the effects of choline supplementation during pregnancy are complex and expensive, often resulting in limited data beyond the immediate postnatal period. Conducting extensive longitudinal studies that follow mothers and children for many years can be resource-intensive. Maintaining participant involvement over extended periods is challenging, limiting the availability of data on the lasting effects of choline supplementation. Despite these challenges, long-term studies are essential for understanding the enduring impact of choline on maternal and child health [41].

Ethics and randomized controlled trials: Conducting RCTs in pregnant women raises ethical concerns, particularly when examining potential risks and benefits. Randomizing pregnant women to a placebo group can be ethically challenging, which limits the ability to establish causal relationships. Ethical considerations and the potential risks to mothers and fetuses often require using non-randomized study designs. This can make it more challenging to draw definitive conclusions about the effects of choline supplementation, emphasizing the importance of complementary research approaches [42].

Confounding factors: Dietary and lifestyle factors, such as maternal smoking, alcohol consumption, and socioeconomic status, can confound the relationship between choline supplementation and pregnancy outcomes, making it challenging to isolate choline's effects. Pregnant women's lifestyles and dietary choices can introduce confounding variables that complicate the interpretation of study results. These factors may independently influence pregnancy outcomes and can be challenging to account for in research designs, necessitating sophisticated statistical methods to control for confounding [43].

## Conclusions

In conclusion, the review of choline supplementation during pregnancy underscores this essential nutrient's vital role in maternal and fetal health. Choline's involvement in neural tube formation, brain development, and preventing complications such as gestational diabetes and fatty liver disease highlights its significance in prenatal care. While research has shown promising potential for choline supplementation to reduce the risk of NTDs, enhance cognitive development in offspring, and improve maternal health, challenges and limitations in study methodology and ethical considerations persist. To address these issues, there is a clear call for increased awareness campaigns to inform pregnant women about the importance of choline, coupled with rigorous research efforts to investigate further the optimal timing, dosage, and long-term effects of supplementation. Ethical guidelines must be followed to safeguard pregnant women's and their offspring's well-being, while policymakers should consider incorporating choline recommendations into official dietary guidelines. Achieving equity in access to choline supplements and knowledge is essential to ensure that all expectant mothers can make informed choices, ultimately contributing to healthier pregnancies and improved outcomes for the next generation.
